# Recent Progress of Induced Spermatogenesis In Vitro

**DOI:** 10.3390/ijms25158524

**Published:** 2024-08-05

**Authors:** Siqi Liu, Jiang Wu, Xin Zhao, Meng Yu, Masayasu Taniguchi, Huimingda Bao, Kai Kang

**Affiliations:** 1 College of Coastal Agriculture Science, Guangdong Ocean University, Zhanjiang 524088, China; 2112204105@stu.gdou.edu.cn (S.L.); wuj@gdou.edu.cn (J.W.);; 2 State Key Laboratory for Mechanical Behavior of Materials, Xi’an Jiaotong University, Xi’an 710049, China; 3 Department of Veterinary Medicine, Yamaguchi University, Yamaguchi 753-0841, Japan

**Keywords:** spermatogenesis, proliferation and differentiation, extracellular matrix, three-dimensional culture system, in vitro induction

## Abstract

Sperm, a crucial gamete for reproduction in sexual reproduction, is generated through the proliferation, differentiation, and morphological transformations of spermatogonial stem cells within the specialized microenvironment of the testes. Replicating this environment artificially presents challenges. However, interdisciplinary advancements in physics, materials science, and cell engineering have facilitated the utilization of innovative materials, technologies, and structures for inducing in vitro sperm production. This article offers a comprehensive overview of research progress on inducing in vitro sperm production by categorizing techniques into two major systems based on matrix-based and non-matrix-based approaches, respectively. Detailed discussions are provided for both types of technology systems through comparisons of their similarities and differences, as well as research advancements. The aim is to provide researchers in this field with a comprehensive panoramic view while presenting our own perspectives and prospects.

## 1. Introduction

Sperm is an essential germ cell for reproduction in bisexual organisms, originating from the proliferation, differentiation, and morphological changes of spermatogonial stem cells (SSCs) within the specialized microenvironment of the testes [1]. Through meiosis facilitated by the synaptonemal complex, sperm carry half of the male genome and undergo chromosome recombination induced by this complex. This distinct process distinguishes them from their parent organism and enables sperm to play a diverse role in promoting genetic diversity during sexual reproduction. However, the delicate microenvironment of the testes often experiences unstable or destructive alterations due to factors such as diseases, drugs, injuries, special physical ion therapy, and extreme environments [2,3,4]. These detrimental influences significantly impact normal sperm development. Therefore, cellular biology and cell engineering techniques have been increasingly employed in research on artificially controlled cultivation and acquisition of high-quality gametes-sperm under these adverse circumstances before or during their occurrence [5].

The induction of spermatozoa development outside the body is an exceedingly intricate and challenging biological process, as it poses difficulties in artificially replicating the specialized “niche” environment required for spermatogenesis [6]. This environment comprises supportive cells within the testes and is formed through coordinated interactions among various peripheral cells surrounding seminiferous tubules, collectively known as the “blood–testis barrier” structure [7]. The successful induction of sperm development in vitro requires the simulation of this specialized microenvironment, along with the provision of essential cytokines and nutrients to support cell proliferation, differentiation, and morphological transition from spermatogonium to mature sperm. Researchers have dedicated their efforts towards developing more convenient and efficient systems for exogenous induction of spermatozoa development, progressing from early in situ cultivation of testicular tissue to artificial construction of niche environments with increasingly remarkable achievements [6].

The article provides a comprehensive overview of the research progress in the field of in vitro sperm induction. It categorizes the techniques into two major systems based on matrix-based and non-matrix-based approaches and offers detailed discussions on these systems, including matrix formulation, culture medium formulation, detection markers or cell characteristics, and whether sperm cells or offspring are generated. By comparing the similarities and differences among various technical systems and their research progress, it aims to offer researchers in this field a comprehensive and detailed panorama while presenting our perspectives and prospects.

## 2. The Progress of Spermatogenesis

**Figure 1:**
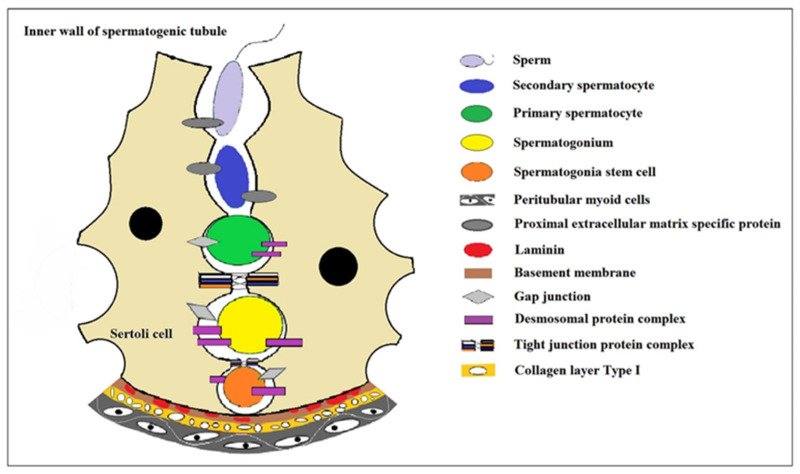
The niche environment during spermatocyte development. In the convoluted seminiferous ducts of the testicles, there are only Sertoli cells and spermatogonium cells (spermatogonium cells are divided into type A_1_, type A_2_, type A_3_, type A_4_, and type B spermatogonium cells according to the sequence they formed after division). A series of cell biological changes in spermatogonium cells is carried out in the niche microenvironment where the Sertoli cells formed. Modified from Article [1].

Sperm production is a complex and coordinated process in which germ cells undergo proliferation and differentiation within the seminiferous tubules of the testes to generate haploid, motile spermatozoa. Thousands of sperm are produced daily within the male gonads or testes [8,9]. Testicular morphogenesis in mammals begins shortly after birth and continues until puberty, culminating in the completion of the initial round of spermatogenesis that establishes a framework for continuous sperm production persisting into old age. Spermatogenesis can be categorized into three stages: spermatocytogenesis involving mitotic cell division to amplify output and generate stem cells as well as primary spermatocytes; meiosis entailing replication and genetic material exchange along with two rounds of cell division to reduce chromosome number to haploid state while producing four spermatids; and spermiogenesis during which spherical spermatids differentiate into mature sperm without further division, subsequently being released from the luminal surface by supporting cells [10,11,12,13,14]. Spermatozoa harbor complete sets of genetic information for their respective species, making them vital carriers for transmitting genetic data. Therefore, normal sperm development plays an indispensable role in species preservation and maintenance of genetic diversity. The entire process from spermatogonia to mature sperm takes place within seminiferous tubules where basal cells, luminal compartments, and tubular spaces constitute crucial microenvironments governing proliferation, differentiation, and survival during gametogenesis (Figure 1). The architecture of these microenvironments facilitates direct interaction between germ cells with somatic support cells as well as signaling cascades dictating germ cell fate [15].

**Figure 2:**
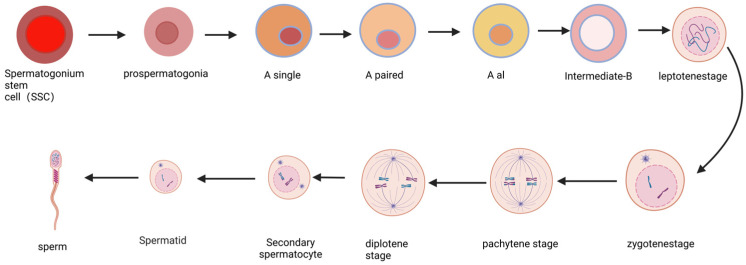
The process of spermatogonial differentiation. The process of spermatogonial differentiation involves the sequential development of primordial germ cells into prospermatogonocytes, followed by their differentiation into A single, A paired, al, intermediate, and B type spermatogonocytes. Subsequently, these spermatogonocytes undergo meiosis to form early spermatogonia in the leptotene and zygotene stages. Finally, late spermatogonium cells progress through pachytene and diplotene to differentiate into secondary spermatogonia before ultimately maturing into sperm.

SSCs are a population of reproductive stem cells residing in the testes of male animals. They represent a type of unipotent stem cell with a remarkable capacity for self-renewal and differentiation, serving as the fundamental basis for sperm production and male reproduction [6]. Due to these extraordinary abilities, SSCs can be perpetually maintained and continuously generate sperm throughout an individual’s lifetime, thereby facilitating the transmission of genetic information to subsequent generations. Consequently, SSCs play an indispensable role in male fertility [16]. Notably scarce, SSCs are estimated to have a concentration of 1/3000 cells within adult mouse testes [10]. These unique cells exist within a specialized microenvironment known as the niche, which governs testicular homeostasis by precisely balancing the self-renewal and differentiation processes of SSCs (Figure 2). The stem cell niche comprises cellular and extracellular matrix components along with local soluble factors that intricately regulate cell fate proximal to the stem cells [17]. The SSC niche assumes a critical function in preserving the potentiality of these stem cells by providing external factors that sustain both self-renewal and differentiation processes [18].

The primordial germ cells are primarily classified into three types: A-type, intermediate-type, and B-type primordial germ cells [18]. The A-type group includes Asingle (As), Apaired (Apr), Aaligned (Aal), A1, A2, A3, and A4. SSCs are typically regarded as As primordial germ cells, representing the most primitive type without intercellular bridges. Upon differentiation of SSCs to generate offspring, spermatogenesis initiates. Apr primordial germ cells commit to further development into sperm rather than self-renewal. Apr primordial germ cells undergo a series of mitotic cell divisions to progress from being an Aal(4) to an Aal(8) and then an Aal(16) before transforming into an A1 primordial germ cell [19]. Subsequently, a series of proliferative divisions leads to the formation of the intermediate stage, with matured forms being the primary and secondary spermatocytes that ultimately produce haploid spermatozoa [10] (Figure 2). In this model, all more advanced types of germline stem cells beyond SSCs (As) are considered differentiated germline cells. Recent studies have demonstrated that Apr and Al exhibit stem cell activity [20,21].

## 3. The Induced Spermatogenesis In Vitro

### 3.1. Scaffold-Based Induced Spermatogenesis In Vitro

#### 3.1.1. Cell-Scaffold-Based Induced Spermatogenesis

Sertoli cells, fibroblasts, and mesenchymal stem cells are commonly employed as cellular scaffolds. Sertoli cells (SCs) represent the principal somatic cells within the SSC niche, providing support for sperm cell morphogenesis through intercellular interactions and secretion of biochemical components such as lactate, cytokines, and hormones. In addition to their mechanical and nutritional roles, SCs also establish an immune-protective environment for germ cells via the blood–testis barrier. Support cells are considered the primary targets of testosterone signaling. Within these support cells, testosterone signals can directly influence gene expression or activate kinases that regulate processes essential for spermatogenesis progression. During spermatogenesis, support cells stimulate SSC self-renewal and promote SSC differentiation while regulating the meiosis of spermatogonia and the transformation from round spermatids to mature spermatozoa [6]. Studies have demonstrated that the overall efficiency and effectiveness of spermatogenesis largely depend on the presence of a supportive cell-SSC niche [22]. Zhang et al. showed that utilizing neonatal pig autologous support cells as feeders had superior promoting effects on SSCs compared to using SIM mouse embryonic-derived STO cell line or adult pig support cells with upregulation observed in differentiation markers c-kit and Stra8 expression [23]. Khajavi et al. co-cultured germinal cells with supporting cells along with collagen protein, resulting in increased expression of meiotic marker SCP3 and post-meiotic markers Crem and TTF1 alongside a positive impact on extensive clone formation, including an increased number of clones with larger diameter [24].

Currently, mouse embryonic fibroblasts (mEF) are the most commonly utilized fibroblasts in cell culture. Kanatsu et al. demonstrated that serum-free conditions on MEFs can support the expansion of mouse primordial germ cells, characterized by strong expression of EpCAM, CD9, α6- and β1-integrin, and weak expression of c-kit [25]. Tiptanavattana et al. successfully cultured cat primordial germ cells on MEFs for 57 days, showing expression of SSC marker GFRα-1 and germ cell marker DDX-4 but no detectable expression of differentiation gene c-kit [26]. Shi et al. achieved a one-month cultivation of mouse primordial germ cells on MEFs and observed the presence of Oct4 and Sox2 expressions in the cultured SSCs, which are crucial factors for self-renewal [27]. Oatley et al., using bovine fetal fibroblasts (BFF) as feeder layers, maintained undifferentiated bovine primordial germ cells for at least one month with expressions of ZBTB16 and LIN28 [28].

The mesenchymal stem cells can be categorized into two types: bone marrow-derived mesenchymal stem cells (BM-MSCs) and human placental mesenchymal stem cells (hPMSCs). Several potential mechanisms for the recovery of testicular function during MSC-induced tissue regeneration have been identified [29]: (1) The participation of MSCs in inhibiting anti-sperm antibodies (ASA) is possible. (2) Mesenchymal stem cells have the ability to decrease cell apoptosis, thereby reducing infertility factors. (3) The presence of mesenchymal stem cells can lead to a reduction in oxidative stress levels. (4) Mesenchymal stem cells are capable of stimulating testosterone production and differentiating into Leydig cells. (5) The differentiation potential of mesenchymal stem cells allows them to develop into specific target cell types. (6) Transplanted MSCs secrete growth factors like bone morphogenetic proteins (BMPs) and transforming growth factor-beta (TGF-β), which act as inducers for male germ cell development, thus facilitating the restoration of receptor cell function. (7) Mesenchymal stem cells interact with endogenous cells to restore impaired cellular function. (8) Mesenchymal stem cells reverse the dysregulation of glycolysis and gluconeogenesis in sperm by modulating the Akt/glycogen synthase kinase-3 (GSK3) axis. (9) Mesenchymal stem cells have the ability to modify the expression of specific miRNAs associated with sperm development and their target genes.

HPMSCs ameliorate chemotherapy-induced testicular damage by mitigating cell apoptosis and oxidative stress, as well as promoting autophagy [30]. BM-MSCs, sharing similar embryonic origin, differentiation potential, and immune regulatory capabilities with supporting cells, play a pivotal role in regulating spermatogenesis through their proliferation and gene expression profile. The direct injection of allogeneic BM-MSCs into the testes has been shown to enhance the population of spermatogonial cells and restore the testicular microenvironment in infertile rats [31]. There are two hypotheses regarding how BM-MSCs promote sperm production: firstly, they may secrete substances that facilitate sperm production; secondly, they themselves can differentiate into sperm [29]. Onen et al. successfully cultured germ stem cells using BM-MSCs for 42 days, resulting in improved in vitro sperm production characterized by differentiated c-Kit (+) germ cells, VASA (+) total reproductive cells, including primary and mature spermatozoa, as well as meiotic division cells [32] (All of the above is briefly summarized in Table 1).

The fabrication of cell scaffolds typically relies on cell lines, which differ from primary cells and cells in the body, potentially leading to biased outcomes. Prior to scaffold formation, cell line cells must undergo pre-culturing for stabilization. However, this process may introduce contamination and variations due to different individuals and batches of reagents used for cell culture, resulting in noticeable discrepancies between experimental results.

#### 3.1.2. Biomaterial-Based Scaffold Induced Spermatogenesis

**Figure 3:**
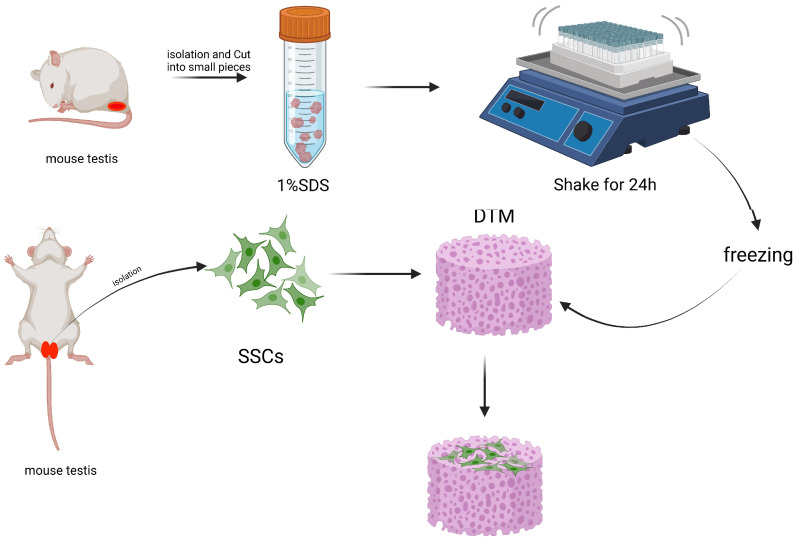
Spermatogenesis was induced using a biomaterial-based scaffold. Mouse testicles were isolated, thoroughly washed with PBS solution, and then cut into small pieces. These tissue fragments were treated with 1% sodium dodecyl sulfate (SDS) as a decellularization solution and incubated for 24 h under shaking conditions. Subsequently, the slices were immersed in PBS for 2 h to remove the detergent. The resulting decellularized tissues were disinfected with 70% ethanol for 1 h, followed by washing and soaking in PBS for an additional 2 h before being frozen for future use. Prior to usage, the decellularized tissues were prepared into a gel matrix and used to culture mouse spermatogonium cells.

The most commonly used testicular scaffold is a decellularized testicular matrix (DTM). DTM is obtained by treating testicular tissue with SDS to remove cells while preserving the typical three-dimensional structure and major components of the natural tissue scaffold (Figure 3), including type I and IV collagen, fibronectin, laminin, and glycosaminoglycans [6]. Moreover, proteomic analysis conducted by Baert revealed the intricate composition of DTM with the presence of numerous other extracellular matrix proteins [33]. Additionally, Noghani et al. discovered that culturing mouse SSCs on DTM hydrogel scaffolds containing the stimulant D-serine led to an upregulation in Plzf expression levels. This finding suggests that DTM hydrogel scaffolds are suitable for SSC culture and promote their proliferation [34]. In another study by Noghani et al., it was observed that culturing mouse SSCs on DTM hydrogel scaffolds containing D-serine significantly increased the expression of pre-meiotic gene Plzf, meiotic gene Sycp3, and post-meiotic gene Tnp1. These results indicate that DTM hydrogels can enhance both proliferation and differentiation of SSCs [35].

Ashouri et al. discovered that human spermatogonial cells cultured on a decellularized testicular matrix (DTM) derived from sheep testes exhibited significantly enhanced expression of pre-meiotic genes OCT4 and PLZF, meiotic genes SCP3 and Boule, as well as post-meiotic genes Crem and Protamine2 compared to the two-dimensional (2D) group after 6 weeks of culture [36]. Moreover, the expression of differentiation genes increased with prolonged culture time. Bashiri et al. utilized sheep DTM as an ink for 3D printing hydrogel scaffolds and cultivated mouse testicular cells on them. They observed improved cell viability and engraftment capacity, along with increased expression of pre-meiotic markers Plzf, Gfrα1, and Id4. Furthermore, they found that spermatogonial cells could differentiate into sperm-like cells on the DTM scaffold [37]. Ashouri et al. isolated human spermatogonial cells and cultured them on sheep DTM. Compared to the 2D group, the expression levels of pre-meiotic genes OCT4 and Plzf, meiotic genes SCP3 and BOULE, as well as post-meiotic genes CREM and Protamine2 were significantly upregulated in cells cultured on DTM [38].

Majidi et al. cultured isolated mouse SSCs on DTM, and after 8 weeks of cultivation, there was minimal alteration in the expression level of the Plzf gene, while the expression level of the Sycp3 gene exhibited a significant increase [39]. Rahbar et al. cultivated mouse SSCs with attached testicular fragments on DTM and observed an upregulation in Plzf, miR-10b, and TGF-β genes expression, along with a downregulation in caspase-3 expression. Subsequently, these cultured germ cells were transplanted into busulfan-treated mice testes. After two weeks, the successful homing of transplanted cells was confirmed by the presence of DiI-positive cells adhered to the basement membrane at the basal region of seminiferous tubules [40]. Rahmani et al. performed transplantation of germ cells cultivated on DTM into busulfan-treated mouse testes and later sacrificed these mice for analysis. They observed a significant increase in host testes’ expression levels of Plzf, Thy1, Vasa, and Gfra1 genes, indicating successful colonization of SSCs within their niche [41].

Non-testicular derived biological scaffolds, such as platelet-rich plasma (PRP) and collagen gel, have been utilized in various studies. Khadivi et al. developed a hydrogel scaffold by mixing PRP with calcium chloride at a ratio of 9:1 and incubating it under specific conditions. The PRP scaffold was compared to the 2D group in terms of its impact on the proliferation of SSCs. The findings revealed that the expression levels of GFRa1 and c-KIT significantly increased in the PRP scaffold group compared to other groups, leading to a significant increase in clone numbers [42]. In another study conducted by Lee et al., rat tail tendon-derived collagen gel was combined with DMEM/F12 medium, serum, and human testicular cells for culture purposes. After 12 days of cultivation, round spermatogonial cells were observed, indicating that collagen fibers can effectively promote germ cell differentiation in vitro [9] (All of the above is briefly summarized in Table 2).

However, due to the inherent variability in the production process of DTM and the differences in age and condition among donors, it is inevitable that each batch of DTM will exhibit certain variations, thereby leading to discrepancies in experimental outcomes between different groups.

#### 3.1.3. Non-Biomaterial-Based Scaffold Induced Spermatogenesis

The most commonly utilized organic scaffold is derived from agarose. Park et al. conducted a study where pig SSCs were cultured in a 0.2% (*w*/*v*) agarose 3D hydrogel, and the results demonstrated significant increases in transcript levels of NANOG, EPCAM, UCHL1, GFRA1, and Plzf. Additionally, there were notable elevations in protein levels of Plzf, OCT4, SOX2, and TRA-1-81. Transcription of OCT4 and THY1 was upregulated, while the translation of NANOG and TRA-1-60 was upregulated as well. Furthermore, the transcription level of the germ cell differentiation marker C-KIT exhibited significant downregulation. These findings suggest that compared to a 2D culture microenvironment, a three-dimensional (3D) culture microenvironment can more effectively sustain the self-renewal of pig SSCs [43].

With the advancement of technology, SACS and MCS scaffolds have gained popularity. SACS (soft-agar culture systems) are fabricated using varying concentrations of agarose. Gholami et al. discovered a significant increase in both the number and size of cloned SSCs cultured on SACS, accompanied by higher expression levels of Scp3 and Integrinα6. SACS can effectively regulate the microenvironment for cell clone proliferation and differentiation [15]. MCS (methylcellulose culture systems), on the other hand, are composed of methylcellulose. AbuMadighem et al., utilizing MCS, successfully cultivated cells isolated from busulfan-treated mouse seminiferous tubules; after 4 weeks of cultivation, they observed an augmentation in clone numbers along with increased expression of CD9, VASA, CREM, BOULE, and ACROSIN proteins as well as the presence of sperm-like cells [44]. Huleihel et al., pioneering a 3D MCS culture system for undifferentiated A-type spermatogonia harvested from juvenile rhesus monkey testes, demonstrated their survival under in vitro conditions and commitment to differentiation pathways; after 30 days in culture, they observed expression of meiotic genes VASA, SALL4, and GFR-α1 alongside post-meiotic genes CREM-1and acrosin [45]. Abofoul et al., through their research, found that spermatogonial cells cultured in MCS expressed pre-meiotic markers VASA, c-KIT, GFRa1, CD-9, α -6 -Integrin, OCT4, Plzf; meiotic markers CREM-1, LDH, BOULE; post-meiotic markers protamine, acrosin. This indicates that spermatogonial cells possess the ability to proliferate and differentiate within an in vitro culture system [46].

Various hydrogels and nanofiber materials have been increasingly utilized for the cultivation of SSCs. Hemadi et al. discovered that culturing SSCs on alginate hydrogel resulted in an augmented cloning capacity and sustained SSC morphology for over 60 days, with observed expression of Oct4, Sox2, Nanog, Nanos2, Bcl6b, and Plzf genes [47]. Eslahi et al. employed a poly L-lactic acid nanofiber scaffold (PLLA) as a supportive structure and demonstrated through RT-PCR the expression of specific genes Plzf, Oct4, GFRα-1, VASA, Itgα6, Itgβ1, and c-Kit involved in germ cell differentiation in cultured spermatogonial cells. This suggests that PLLA can promote the proliferation and differentiation of SSCs in vitro [48]. Ziloochi et al. cultivated SSCs on an agar/polyvinyl alcohol nanofiber scaffold (agar/PVA), resulting in a significant reduction of pre-meiotic markers ID-4 and GFRα-1 while increasing post-meiotic markers SYCP-3 and Tektin 1/TEKT-1 expression levels. This scaffold enhanced at least a six-fold increase in the differentiation rate from mouse SSCs to meiosis and post-meiosis cells [49]. Bashiri et al., utilizing an electrospun polycaprolactone/gelatin nanocomposite scaffold (electrospun PCL/gelatin), cultivated spermatogonial cells, leading to an increase in their quantity along with significantly higher expression levels of Plzf gene while c-kit gene expression decreased noticeably. The nanocomposite scaffold provided suitable capability for self-renewal of human spermatogonial cells [50].

With further research, it has been discovered that the scaffold for cultivating SSCs can be composed of various materials. Jabari utilized an agarose and laminin-coated protein scaffold to successfully cultivate human spermatogonial cells, resulting in the presence of Plzf, SCP3, PRM2, Acrosin positive cells as well as sperm-like and elongated sperm cells [51]. In another experiment conducted by Jabari et al., a scaffold was created using SACS along with laminin-coated protein and supporting cells to cultivate SSCs, which exhibited expression of Plzf, α6-Integrin, Bcl2, c-KIT genes [52]. Veisi et al. co-cultivated mouse spermatogonial cells with alginate hydrogel and supporting cells; this led to significantly increased levels of integrin alpha-6, integrin beta-1, Nanog, Plzf, Thy-1, Oct4a, and Bcl2 expression. This scaffold effectively promoted proliferation and maintained the self-renewal capacity of SSCs while improving the efficiency of SSC transplantation [22]. Zhao et al. cultivated purified pig SSCs on poly-L-lysine (PLL) coated dishes along with laminin coating for 28 days without compromising their undifferentiated germ cell phenotype [53]. He et al. cultured mouse spermatogonial cells on laminin-coated protein (LCP) and poly-L-lysine (PLL), demonstrating expression of VASA, GPR125, Uchl1, GFR-A1, and DAZL genes indicating that LCP and PLL-based in vitro culture system is efficient in long-term maintenance of stable SSCs with self-renewal ability [54]. Xu et al. utilized a gelatin-based hydrogel known as Dynamic gelatin-based hydrogels to mimic the inherent structural dynamics of ECM for the cultivation of primordial germ cells. Following cultivation, notable expression of pluripotency markers such as NANOG and OCT3/4 was observed, along with the presence of nestin-positive, alpha-fetoprotein-positive, and alpha-SMA-positive cells representing differentiated cells from all three germ layers. These findings indicate that mESCs obtained after 2 months of 3D cultivation in GelCD hydrogel possess functional pluripotency. GelCD hydrogel serves as an effective 3D cultivation platform supporting the long-term proliferation and self-renewal of mESCs [55]. Poels et al., on the other hand, cultured cryopreserved mouse testicular tissue in agarose hydrogel containing VEGF nanoparticles and demonstrated maintenance of seminiferous tubule integrity with the presence of Plzf- and KI67-positive cells. This gel formulation can enhance the primordial germ cell recovery rate [56] (All of the above is briefly summarized in Table 3).

The gel is not capable of effectively simulating the niche environment necessary for the growth of SSCs, and additional supplementation is required to provide various factors essential for cell growth. The types of growth factors provided by humans differ from those present in the natural niche environment, thus rendering in vitro culture models potentially incongruent with expectations.

#### 3.1.4. Organ Culture Method Induced Spermatogenesis

**Figure 4:**
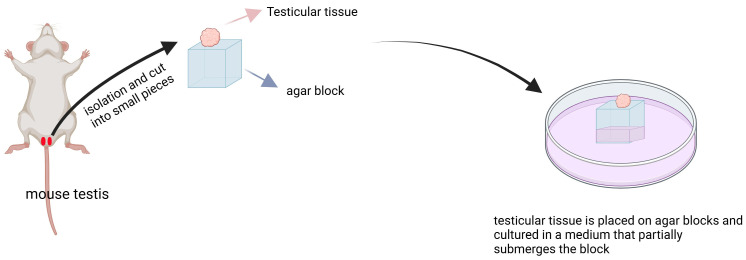
The organ culture method was employed to induce spermatogenesis in mice testicles. The testicles were dissected and cut into small 1cm pieces, which were then placed onto a gel block. Subsequently, the gel block was immersed in a petri dish containing culture medium, with half of the gel block submerged.

The organ culture method involves the preparation of agar blocks using a mixture of agar and water. Subsequently, testicular tissue is placed on these agar blocks and cultured in a medium that partially submerges the block (Figure 4). Gholami et al. utilized this method to culture mouse spermatogonial cells and observed the presence of Plzf, SCP3, and KI67-positive cells on the agar block [57]. In another experiment by Gholami et al., they also employed the organ culture method to cultivate mouse spermatogonial cells and noted an increased expression of Plzf, Integrin α6, Scp3, Mvh [58], indicating that this technique can facilitate proliferation and differentiation of spermatogonial cells in vitro. Kojima et al. used the organ culture method to cultivate mouse spermatogonial cells and witnessed more than a 3-fold increase in testicular tissue volume after 12 days of cultivation along with GFP expression and production of round or elongated spermatozoa [59]. Sato et al. employed the organ culture method to cultivate mouse spermatogonial cells, resulting in the observation of GFP-positive cells as well as round spermatozoa formation, thereby demonstrating its effectiveness for culturing mouse testicular tissue and supporting in vitro sperm development [60]. Reda et al., through the utilization of the organ culture method, successfully cultured rat testicular tissue for 52 days, which exhibited expression of Acrosin and Crem positive proteins; testosterone production was observed after 3 days, while the addition of adipose tissue from epididymis led to spontaneous contraction of cultured seminiferous tubules after 21 days [12]. Matsumura et al. demonstrated that the utilization of organ culture techniques can effectively induce in vitro spermatogenesis from mouse germ cells, leading to the development of mature spermatozoa that can be sustained for a duration exceeding 70 days within the cultured tissue [61]. Sato et al. successfully cultivated mouse germ cells utilizing organ culture methods, wherein they observed GFP expression after 18–30 days of cultivation, which persisted for a period ranging from 15–45 days or even longer. Furthermore, they obtained viable mouse sperm capable of generating healthy offspring through intracytoplasmic sperm injection [62] (All of the above is briefly summarized in Table 4).

At present, there are few studies on the specific role of testicular organ culture systems in vitro spermatogenesis, including whether somatic cells develop normally and whether they can interact with germ cells and correctly regulate spermatogenesis.

### 3.2. Non-Scaffold-Based Induced Spermatogenesis In Vitro

#### 3.2.1. RA-Induced Spermatogenesis

Retinoic acid (RA) is a metabolite derived from vitamin A [63]. Upon binding to its high-affinity RA receptor (RAR), RA exerts regulatory effects on transcription by modulating the RA response elements in target gene promoters. RAR comprises three isoforms, namely RARα, RARβ, and RARγ. In neonatal, adolescent, and adult mammalian testes, RARα predominantly localizes in the supporting cells of the testes, while RARγ is primarily expressed in differentiating spermatogonia. The main role of RA lies in regulating spermatogonial differentiation through its interaction with RAR [64]. Insufficient levels of RA lead to an increase in SSC values in newborn mouse testes [5]. Spermatogonial differentiation critically relies on the presence of RA [65]. Chronic vitamin A deficiency or administration of an RA receptor antagonist, such as WIN18446, hampers spermatogenesis at the undifferentiated (Aal) stage, ultimately leading to azoospermia and infertility in mice. Fertility can be restored by promoting the maturation of spermatogonocytes from Aal type to A1 type through supplementation with vitamin A or exogenous RA [66].

In Sertoli cells, retinoic acid (RA) enhances the expression of Kit ligand (Kit receptor) and bone morphogenetic protein 4 (BMP4), which in turn inhibits the expression of glial cell line-derived neurotrophic factor (GDNF). In undifferentiated spermatogonocytes, RA binds to RARγ to stimulate the expression of Kit and Stra8 [19]. The signaling pathway mediated by RA promotes spermatogonial differentiation until meiosis initiation occurs. RA plays a crucial role in inducing the differentiation of undifferentiated spermatogonia and guiding their progression into meiosis [67]. Zhao et al. utilized RA and N2B27 medium to induce porcine spermatogonocytes and flow cytometry analysis revealed a distinct haploid peak value after induction in the 5th and 10th generations, indicating successful induction of meiosis in cultured SSCs [68]. Sanjo et al. supplemented RA, gonadal hormones, and lipids to induce spermatogenesis in spermatogonia; they observed a positive signal for γ-H2AX that was consistent with leptotene-stage cells, but no sycp1-positive cells were detected, suggesting that meiotic progression did not reach the pachytene stage. The addition of exogenous RA or retinol alone can effectively trigger meiosis initiation in undifferentiated spermatogonia [67] (All of the above is briefly summarizedin Table 5).

#### 3.2.2. Hormones or Growth Factors Induced Spermatogenesis

Exogenous gonadotropins have the ability to induce spermatogenesis in men with hypogonadism characterized by low levels of gonadotropins [69]. Miura et al. conducted tissue section staining and discovered that Human Chorionic Gonadotrophin (hCG) could effectively stimulate spermatogenesis at all stages in Japanese eel cultured in vitro. Sperm cells and sperm were observed in testes cultured for 21 and 24 days, respectively. Furthermore, it has been found that the action of this gonadotropin may be mediated by interstitial cells and supporting cells [70]. Nader et al. successfully cultivated Japanese eel spermatogonium cells using a combination of 11-ketotestosterone (11-KT) and RHIGF-1, resulting in complete spermatogenesis. However, when RHIGF-1 was used alone, germ cell proliferation was not observed [71]. In a detailed comparison between 11-KT-induced Japanese eel spermatogenesis in vitro and in vivo spermatogenesis induced by a single injection of hCG, Miura et al. concluded that while 11-KT had a promoting effect on spermatogenesis, its efficacy was significantly weaker compared to the natural process occurring within living organisms [72].

Deng et al. discovered that melatonin supplementation significantly enhanced the differentiation of cultured Suffolk sheep SSCs into haploid germinal cells, resulting in a twofold increase in the rate of differentiation into sperm compared to the group without supplementation. Additionally, post-meiosis expression of marker genes Dnmt3a and Bcl-2 was upregulated [73]. Navid et al. also observed that supplementation with 100 μM melatonin in cultured mouse SSCs could significantly promote SSC proliferation, leading to elevated levels of Id4 and Plzf. However, there were no significant differences in C-kit levels compared to the control group [74]. Yang et al. found that increased autophagy can down-regulate the expression of genes responsible for homologous recombination, which is the primary pathway for repairing double-strand breaks (DSBs) during meiosis. This suggests that CdSe/ZnS QDs-induced spermatogenesis damage is mediated by autophagy. The use of an autophagy inhibitor (3-MA) can restore mouse spermatocyte DSB repair, prevent spermatocyte apoptosis, and restore spermatogenesis [75].

**Table 5:** Non-scaffold-based induced spermatogenesis.

Source of SSCs	Culture Substrate of SSCs	Markers of SSCs	Whether to Produce Offspring of SSCs	Reference of SSCs
Sheep	medium	The expression of post-meiosis marker genes Dnmt3a and Bcl-2 was upregulated	Sperm-like cell	[73]
Mice	medium	Id4 and Plzf levels were elevated, but C-kit levels were not significantly different compared with controls	unresearched	[74]
Mice	medium	The expression of GFP was enhanced	Early-round sperm cells and late-round sperm cells were seen	[67]
Pig	STO-containing medium	UCHL1, CDH1, and OCT4 positive cells showed obvious haploid peaks by flow cytometry	unresearched	[68]

## 4. Conclusions and Prospects

The culture system without animal-derived components and substrates and with a clear composition is an imperative future trend. The utilization of animal-derived components, particularly the ambiguous and inconsistent ones like sera, has introduced numerous uncertainties in establishing a stable in vitro-induced spermatogenesis system. Consequently, various research groups have proposed different systems for in vitro-induced spermatogenesis [67,74]. This lack of consistency makes it challenging to establish a unified and stable technical system for artificial induction and its subsequent transformation and application, thereby impeding the progress of this field. Henceforth, researchers should collaborate across interdisciplinary domains such as biophysics, biochemistry, and molecular biology to develop a more reliable and efficient culture system devoid of animal sources or matrices.

Non-animal-derived or structurally transparent artificial matrix culture systems represent the predominant direction in current and future research. Within the unique environment of spermatogenesis, which relies on testicular Sertoli cells and is regulated by various peripheral cells within the seminiferous tubules, diploid germ cells undergo cell proliferation, differentiation, and morphological changes to generate haploid spermatozoa [22,23]. The interface between these supporting cells and germ/sperm cells is filled with a substantial amount of extracellular matrix substances synthesized or transported by supporting cells. These biologically active substances and structural matrix components collectively form the niche environment for germ cell spermatogenesis [76,77]. By leveraging advancements in cellular biology and cell engineering technology along with techniques from biophysics, biochemistry, and molecular biology, we are able to construct diverse forms of three-dimensional (3D) in vitro culture systems that mimic all factors (physical, chemical, and biological) present in the niche environment. This has prompted researchers to develop varied, convenient, and efficient in vitro induction systems for sperm production [9,35,42,54]. Moreover, through integrating biochemical molecular biology with materials, science disciplines such as biomaterials and non-biological materials are increasingly being utilized to fabricate these artificial matrix 3D culture systems. This further enhances the focus on inducing sperm production and transforming technological systems.

Additionally, with the advancement of cryopreservation techniques for tissue and cellular vitrification, researchers can employ mature in vitro induction systems to reprogram thawed testicular tissue or cryopreserved spermatogonial cells that were prepared in advance. By utilizing conventional methods such as tissue block and mixed cell culture, they can also efficiently acquire sperm cells. Moreover, flow cytometry sorting or immunomagnetic bead selection methods can be utilized to obtain purified sperm cells for downstream research or applications [78,79,80,81,82].

In summary, sperm plays a pivotal role in male reproduction and is derived from the proliferation, differentiation, and transformation of SSCs within a specialized microenvironment in the testes. Although inducing ex vivo sperm development is an intricate and challenging biological process to control, creating an artificial “niche” environment for such development remains a significant challenge. However, with the interdisciplinary advancements in physics, chemistry, materials science, molecular biology, cell biology, and cellular engineering fields, it is inevitable that novel materials, technologies, and structures will be applied to facilitate ex vivo sperm development for the betterment of human reproduction and conservation of animal genetic resources.

## Figures and Tables

**Table 1:** Cell-Scaffold-based induced spermatogenesis.

Source of SSCs	Culture Substrate of SSCs	Markers of SSCs	Whether to Produce Offspring of SSCs	Reference of SSCs
Mice	alginate hydrogel with Sertoli cells	The expressions of integrin alpha-6, integrin beta-1, Nanog, Plzf, Thy-1, Oct4 and Bcl2 were increased, while the expressions of P53, Fas and Bax were decreased	unresearched	[22]
Mice	MEF	EpCAM, CD9, α6- and β1-integrin were strongly expressed, while c-kit was weakly expressed	SSCs cultured in vitro were transplanted into the testis of sterile mice to produce normal, fertile offspring	[25]
Mice	supporting cells along with collagen protein	Meiosis markers SCP3 and post-meiosis markers Crem and TTF1	unresearched	[24]
Mice	MEF	The expressions of Oct4 and Sox2 were detected	unresearched	[27]
Mice	hPMSC	The expression of proliferating genes (PCNA and KI67) increased, and the mRNA levels of apoptotic genes such as γ-H2AX, BRCA1, and PARP1 decreased	unresearched	[30]
Mice	BM-MSC	The proportion of c-Kit (+) differentiated spermatocytes to whole testicular cells was significantly higher in the BM-MSC co-culture group, and the number of SCP3 (+) primary and secondary spermatocytes and Acrosin (+) round spermatocytes at days 14, 28, and 42 were higher	unresearched	[31]
Mice	BM-MSC	C-KIT, VASA	unresearched	[32]
Pig	Porcine Sertoli cell	Differentiation gene C-kit, Stra8	unresearched	[23]
Cat	MEF	The cells expressed SSC marker GFRα-1 and germ cell marker DDX-4 but did not express the differentiation gene c-kit	unresearched	[26]
Bull	BFF	The cells expressed undifferentiated cell markers ZBTB16 and LIN28 and SSC markers GFRA1 and NANOS2	unresearched	[28]

**Table 2:** Biomaterial-Based Scaffold Induced Spermatogenesis.

Source of SSCs	Culture Substrate of SSCs	Markers of SSCs	Whether to Produce Offspring of SSCs	Reference of SSCs
Mice	mice DTM	The expression of Plzf was increased	unresearched	[34]
Mice	DTM	The expressions of Plzf, Sycp3, and Tnp1 were significantly increased	unresearched	[35]
Mice	Ram DTM	The expressions of pre-meiosis markers Plzf, Gfrα1, and Id4 were increased	unresearched	[37]
Mice	Mice DTM	The expression of the Plzf gene did not change much, but the expression of the Sycp3 gene increased significantly	unresearched	[39]
Mice	DTM with epididymosomes	The expressions of Plzf, miR-10b, and TGF-β were increased, while the expression of caspase-3 was decreased. Homing occurred after transplantation	unresearched	[40]
Human	Sheep DTM	Compared with 2D group, SCP3, Boule, Crem and Protamine2 expressions were increased	unresearched	[36]
Human	Sheep DTM	The expressions of pre-meiosis genes OCT4, Plzf, SCP3, BOULE, and post-meiosis genes CREM and Protamine2 were significantly increased	unresearched	[38]
Human	PRP	The expressions of GFRa1 and c-KIT were significantly increased	unresearched	[42]
Human	collagen	The expression of RMP2 increased	unresearched	[9]

**Table 3:** Non-biomaterial-based scaffold-induced spermatogenesis.

Source of SSCs	Culture Substrate of SSCs	Markers of SSCs	Whether to Produce Offspring of SSCs	Reference of SSCs
Pig	agarose	Compared with the 2D microenvironment, the transcription levels of NANOG, EPCAM, UCHL1, GFRA1, and Plzf were significantly increased when cultured in 0.2% (*w*/*v*) agarose 3D hydrogel, and the protein levels of Plzf, OCT4, SOX2, and TRA-1-81 were significantly increased. Transcription of OCT4 and THY1 is upregulated, the transcription level of spermatogonial differentiation marker C-KIT is significantly down-regulated, and the translation of NANOG and TRA-1-60 is upregulated	Undifferentiated porcine SSCs transplanted into recipient mouse testis are still differentiated into sperm.	[43]
Rhesus monkey	MCS	The expression of the meiotic gene VASA, SALL4, and GFR-α1, meiotic gene CREM-1, and post-meiotic gene acrosin were observed after 30 days of culture	unresearched	[45]
Human	SACS	The expression of INTEGRIN α6 and SCP3 were increased in the SACS group, and the number and size of spermatogonial stem cell clones were significantly increased	unresearched	[15]
Human	MCS	The expression of pre-meiosis markers VASA, c-KIT, GFRa1, CD-9, α-6-Integrin, OCT4, Plzf, meiosis markers CREM-1, LDH, BOULE, and post-meiosis markers protamine and acrosin could be detected	unresearched	[40]
Human	PCL/Gelatin nanofibrous scaffolds	The number of spermatogonocytes increased, the expression of the Plzf gene increased significantly, and the expression of the c-Kit gene decreased significantly	unresearched	[50]
Human	agarose and laminin	Plzf, SCP3, PRM2, Acrosin positive cells were observed	Sperm-like cells are seen	[51]
Human	SACS and laminin	The expression of Plzf, α6-Integrin, Bcl2, and c-KIT genes can be seen	unresearched	[52]
Mice	MCS	After 4 weeks of culture, the number of clones increased, and the expressions of CD9, VASA, CREM, BOULE, and ACROSIN increased	Sperm-like cells are seen	[44]
Mice	alginate hydrogel	The expression of Oct4, Sox2, Nanog, Nanos2, Bcl6b and Plzf genes was observed	unresearched	[47]
Mice	PLLA	The expression of spermatogonial specific genes Plzf, Oct4, GFRα-1, VASA, Itgα6, Itgβ1 and germ cell differentiation gene c-Kit was observed	unresearched	[48]
Mice	PVA	Premeiosis markers ID-4 and GFRα-1 were significantly decreased, while the expressions of SYCP-3 Tektin 1 and TEKT-1 were increased after meiosis	unresearched	[49]
Mice	alginate hydrogel with Sertoli cells	The expression levels of integrin alpha-6, integrin beta-1, Nanog, Plzf, Thy-1, Oct4 a and Bcl2 were significantly increased	unresearched	[22]
Mice	PLL and laminin	VASA, GPR125, UCHL1, GFR-A1 and DAZL were expressed	unresearched	[54]
Mice	GelCD hydrogel	Pluripotent markers such as NANOG and OCT3/4 were significantly expressed, and nestin-positive, α-fetoprotein-positive, and α-SMA-positive cells represented differentiated cells from all three blastoderms	unresearched	[55]
Mice		Plzf and KI67 positive cells were observed	unresearched	[56]

**Table 4:** Organ culture method induced spermatogenesis.

Source of SSCs	Culture Substrate of SSCs	Markers of SSCs	Whether to Produce Offspring of SSCs	Reference of SSCs
Mice	agarose	Plzf, SCP3, KI67 positive cells were observed		[57]
Mice	agarose	The expressions of Plzf, Integrin α6, Scp3 and Mvh were increased		[58]
Mice	agarose	The testicular tissue volume increased by more than 3 times, and the expression of GFP was observed	There are round or elongated sperm cells produced	[59]
Mice	agarose	GFP-positive cells were observed	Round sperm cell	[60]
Mice	agarose	Tissue section observation	Round sperm cell	[61]
Mice	agarose	GFP begins to express after 18–30 days of culture and can last 15–45 or even longer	Sperm cell	[62]

## Data Availability

All relevant data are within the paper.
